# Employment Insecurity and Material Deprivation in Families with Children in the Post-Great Recession Period: An Analysis for Spain and Portugal

**DOI:** 10.1007/s10834-023-09905-z

**Published:** 2023-05-14

**Authors:** Antonio L. Pérez-Corral, Amélia Bastos, Sara Falcão Casaca

**Affiliations:** 1 University of Valladolid, Valladolid, Spain; 2 ISEG-School of Economics and Management, Universidade de Lisboa, Lisbon, Portugal; 3 REM - Research in Economics and Mathematics, CEMAPRE, Lisbon, Portugal; 4 SOCIUS/CSG - Lisbon School of Economics & Management, Universidade de Lisboa, Lisbon, Portugal

**Keywords:** Child well-being, Economic crisis, Household poverty, Precarious employment, Unemployment

## Abstract

The aim of this paper is to analyse the relationship between household employment insecurity and the risk of children's exposure to household material deprivation in Spain and Portugal. Specifically, using EU-SILC microdata for 2012, 2016 and 2020, it examines how this relationship evolved during the Post-Great Recession period. Although in both countries there was an improvement in the employment situation of individuals and families after the Great Recession, the main findings reflect an increase in the risk of children's exposure to material deprivation in households where no adults have a secure job. However, there are some differences between the two countries. In the case of Spain, the results seem to indicate that the incidence of household employment insecurity on material deprivation was higher in 2016 and 2020 than in 2012. In Portugal, the increase in the effect of employment insecurity on deprivation seems to have occurred only in 2020, the year the Covid-19 pandemic began.

## Introduction

The economic crisis that began in 2008, also known as the Great Recession (Keeley & Love, 2010), had severe consequences, including the intense destruction of jobs and the impoverishment of the population (Gutiérrez, 2014; Van Gyes & Szekér, 2013). In Europe, the so-called southern countries (Greece, Italy, Portugal, and Spain) were among the most affected. In addition, as a result of the crisis, austerity measures and labour reforms were implemented in these countries, which also had a negative impact on the living conditions of families and increased social inequalities (Gálvez-Muñoz et al., 2013; Zartaloudis, 2014). Consequently, the greater economic and employment precariousness resulting from the recession, together with a slow recovery in the subsequent period, left a large part of the population in a very vulnerable situation at the beginning of the crisis caused by the Covid-19 pandemic (OECD, 2020).

Poverty is a significant threat to family interactions and functioning (Bao & Greder, 2022; Conger et al., 1994; Liu et al., 2022) and consequently the well-being of children (Fanjul, 2014). In fact, according to the empirical evidence available, experiencing poverty during childhood is associated with several disadvantages in adult life, such as having a low educational level, health problems or bad working conditions (see, for example, Duncan et al., 2010, 2012; Lacour & Tissington, 2011; Oshio et al., 2010). Therefore, it is to be expected that the increase in economic difficulties and job precariousness over the last ten years has harmed the future opportunities of many children.

There are several studies for southern European countries that have shown the detrimental effect of the Great Recession on the economic and employment situation of families and on child well-being (e.g., Ayllón, 2017; Chzhen, 2017; D’Agostino et al., 2019; Natali & Saraceno, 2017; Rajmil et al., 2015). However, less information exists of the subsequent recovery period's impact on children. This article aims to contribute to the literature by examining the employment situation of families’ adult members in the Post-Great Recession period and its impact on child well-being. Following the work of Ferrão et al. (2021), we will analyse the risk of exposure of children to household material deprivation, an essential determinant of child well-being. Specifically, we focus on the cases of Spain and Portugal. These two southern European countries share many characteristics and backgrounds, among which the great relevance of the family as a provider of well-being, the labour market segmentation by age, having had a dictatorial regime for a large part of the twentieth century, or not having joined the European Union until the mid-1980s (Gutiérrez, 2014; Karamessini, 2008). Despite their similarities, they also present some differences in terms of family policies and the participation of women in the labour market that may have been determining factors in the vulnerability of families to the increase in job precariousness and unemployment (Alcañiz et al., 2016; Escobedo & Wall, 2015; Tavora, 2012; Wall & Escobedo, 2011). This paper addresses the following research questions: How has the incidence of household employment insecurity on children's risk of exposure to material deprivation evolved after the Great Recession? Has this evolution been similar in Spain and Portugal?

After this introduction, in the next section we describe the evolution of the employment context in Spain and Portugal since the beginning of the Great Recession, review the literature on child poverty, and present the background of the relationship between employment situation and deprivation. The database, variables, and methodology are then described. Subsequently, the results obtained in the analyses are shown. The last section presents the discussion of the main findings and the conclusions.

## Background

### Unemployment and Job Precariousness in Spain and Portugal

**Fig. 1:**
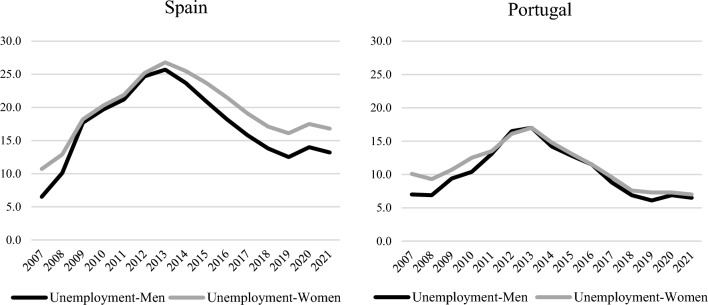
Unemployment rate by sex (% of active population aged 15–64), 2007–2021. *Source* Eurostat (Labour force survey)

The large increase in unemployment was one of the main consequences of the 2008 economic crisis in Spain and Portugal (OECD, 2012). In both countries, the destruction of jobs, especially during the first stage of the crisis, was concentrated in sectors where the presence of men has traditionally been high, such as construction and manufacturing (Addabbo et al., 2015; Ferreira, 2013; González Gago & Segales Kirzner, 2013; Périvier, 2014). Even so, it should also be noted that in the last stage of the recession, the austerity measures of the governments aimed at reducing the budget deficit had a negative impact on employment in the public sector, in which there is greater participation of women. Portugal implemented these austerity measures to meet the conditions of the Troika bailout program (Addabbo et al., 2015). As shown in Fig. 1, during the recession period, unemployment increased more in Spain than in Portugal. This is because the Spanish labour market was not only affected by the global economic crisis but also by the bursting of the construction sector bubble (González Gago & Segales Kirzner, 2013; Malo, 2015). Spain, along with Greece, was the European Union country where the unemployment rate increased the most between 2008 and 2013 (Gutiérrez, 2014).

In response to employment problems, both countries approved a series of reforms to achieve greater flexibility in the labour market (Barroso, 2017). These reforms weakened the position of workers in collective bargaining, facilitated dismissal, increased working hours, and froze increases in the minimum wage. Consequently, the conditions of the Spanish and Portuguese labour market deteriorated, which entailed an increase in working poverty (Addabbo et al., 2015; González Gago & Segales Kirzner, 2013).

In this context of greater precariousness and high unemployment rates, the period after the recession was characterized by a slow recovery, particularly in the Spanish case (OECD, 2018). Regarding the effects of the Covid-19 pandemic, as Fig. 1 shows, in both countries the consequences of the pandemic on unemployment were much less than those of the Great Recession, which is largely due to the measures adopted to retain employment and support enterprises and workers (ILO, 2020). On the other hand, in both countries, average real earnings had not fully recovered from the consequences of the Great Recession when the Covid-19 crisis struck (OECD, 2020).

**Fig. 2:**
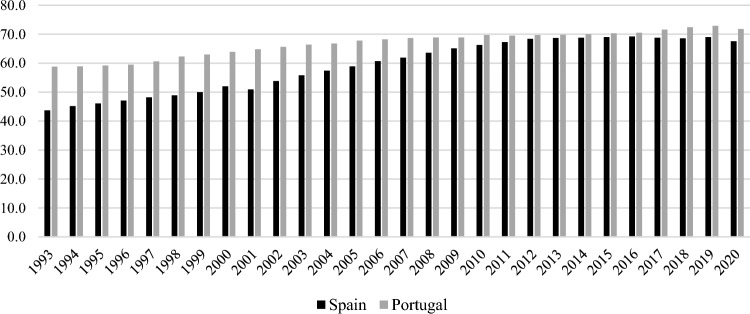
Female activity rate, 1993–2020. *Source* Eurostat (Labour force survey)

Apart from the fact that, in terms of unemployment, the recession of 2008 hit Spain harder than Portugal, there are some differences between these two countries that may be decisive in the lower capacity of Spanish families to face periods of increased unemployment and precarious employment. First, female activity and employment rates have traditionally been much higher in Portugal (Casaca, 2012; Karamessini, 2008; Tavora, 2012). It is because the integration of Portuguese women in the labour market dates back to the 1960s, when emigration and the recruitment of men for the colonial wars led to a sharp decline in the male workforce (André, 1996; Casaca & Damião, 2011; Torres et al., 2004). In the case of Spain, the increase in female activity and employment rates has been more recent (Salido, 2011). Even so, as can be seen in Fig. 2, in the last 20 years the Spanish female activity rate has come quite close to that of Portugal.

Despite the deficiencies in family policies in both countries, the high level of female employment in Portugal may have promoted further development in work-family reconciliation policies there (Escobedo & Wall, 2015; Tavora, 2012). In fact, maternity has a negative impact on the probability of women having a job in Spain, while this detrimental effect is non-existent in Portugal (Casaca & Damião, 2011; Tavora, 2012). Given the participation of women in the labour market in Portugal, families with children may have had greater protection against the labour difficulties of the last decade (Rubery, 2013; Távora & Rodríguez-Modroño, 2018).

### Child Poverty

The employment consequences of the Great Recession increased poverty in families with children in Spain and in Portugal, countries where public family transfers are lower than most European countries (Cantó & Sobas, 2020; Wall et al., 2015). There are several approaches to conceptualizing child poverty, all of which have in common the acceptance of the multidimensional nature of the problem (Pinilla-Roncancio et al., 2021; Saunders & Brown, 2020). In this respect, among the main dimensions considered, the following stand out: income poverty, health, education, behaviour, subjective well-being, and safety (Bradshaw, 2015; Bradshaw et al., 2006; Cho & Yu, 2020; OECD, 2016). In general, much of the previous literature has focused on the economic perspective of child poverty, using income indicators (Bradshaw et al., 2006; OECD, 2009). Studies such as those by Bradshaw (2015) and Treanor (2018) have pointed out the detrimental effect of income poverty on the rest of the dimensions. In the European context, it is very common to identify as poor children those who live in a household with an equivalent disposable income of less than 60% of the national median income (see, for example, Bárcena-Martín et al., 2018; Chzhen, 2017; Chzhen & Bradshaw, 2012). According to Eurostat data for this indicator, 16.8% of children in the European Union were at risk of poverty in 2021. In the specific cases of Spain and Portugal, the percentages were 21.7% and 18.4% respectively. Despite its wide use, income poverty risk indicators as a measure of child poverty can pose problems. For example, these indicators are highly sensitive to changes in the general income level of countries during crisis and recovery stages, they do not take into account household resources such as savings or loans, and they assume that income is shared equally among household members (Bárcena-Martín et al., 2017b; Bradshaw, 2015).

Deprivation gained relevance in the study of poverty in the work of Townsend (1979, 1987). According to this author, individuals experience deprivation when they lack essential material goods or do not participate in the usual activities of their society (Townsend, 1987). Although there is a link between income poverty and deprivation, these two situations are not always simultaneous (Fusco et al., 2011; Landiyanto, 2022). That is, not all income-poor families experience deprivation and not all deprived families are income poor. Many studies for European countries have used deprivation indicators based on the lack of necessary items, especially since the implementation of the Europe 2020 strategy (Guio et al., 2016). Chzhen and Bradshaw (2012) and Chzhen (2014) have used standard household deprivation items to measure deprivation in families with children. Other authors have used specific items of child deprivation (Bárcena-Martín et al., 2017a, 2017b; Chzhen et al., 2018; Guio et al., 2020). However, these items usually use the household as the unit of measurement and, therefore, do not report the specific individual deprivation level of each child. The scarcity of data centred on children has been a major constraint. In a recent study, Ferrão et al. (2021) developed a measure of children's exposure to household material deprivation.

Previous empirical evidence has shown that household deprivation has a greater association with children's overall well-being than income poverty (Bradshaw, 2015; Lau & Bradshaw, 2018; Main & Pople, 2011). These findings reflect that deprivation is a significant determinant of child well-being, so having more studies that analyse the factors that influence it in specific contexts may be very useful for the design of social and family policies.

### Research on the Effect of Employment Problems on Material Deprivation

Lack of employment and precarious employment increases the risk of material deprivation, mainly because these work circumstances affect both the decrease in household financial resources and the increase in the uncertainty of future income (Figari, 2012; Layte et al., 2001). There is extensive literature examining the relationship between employment status and material deprivation. Firstly, some studies use a single general measure of deprivation based on the lack of different types of goods and services. In an analysis for European countries, Figari (2012) found that unemployment and job instability increase the probability of experiencing material hardship. Also for European countries, de Graaf-Zijl and Nolan (2011) found a high risk of material deprivation in households where none of its members works. In a study for the case of Hong Kong, Cheung et al. (2019) showed, among other results, that workers with a temporary job have a greater risk of deprivation than those with a permanent contract. Other authors have focused their attention on specific types of deprivation. Huang et al. (2016) showed that unemployment increases the risk of food insecurity in the United States. Álvares and Amaral (2014) found the same result for Portugal. Ahn and Song (2017) analysed the incidence of unemployment on four types of material hardships for American adults between 50 and 61 years of age. These authors found that, for this subgroup of the population, unemployment is associated with material hardship in the domains of bill-paying, health, and food. However, the results did not show that unemployment affects housing hardship. Eamon and Wu (2011) indicated that single mothers who are underemployed or unemployed have a higher risk of experiencing material difficulties related to bill-paying, health, and housing than those mothers with adequate employment. Furthermore, families in which the mothers are unemployed were also more vulnerable to food deprivation.

There are also studies that analyse the specific material deprivation experienced by children. Although the main objective of these studies was not to examine the relationship between the employment situation of parents and the specific deprivation of children, among their results, they found that lack of employment and precarious employment are associated with a greater risk of child deprivation (see Bárcena-Martín et al., 2017a, 2017b; Chzhen et al., 2018; Guio et al., 2020; Main & Bradshaw, 2014).

One aspect to highlight is that, despite the numerous studies conducted to date on the relationship between employment status and material deprivation, there is scant knowledge of how the employment context of the country can influence this relationship. In the first place, the context of increased job uncertainty in periods of economic crisis, understood as a greater risk of job loss and worse job-search prospects (Ravn & Sterk, 2017), must be taken into account. This increase in job uncertainty mainly affects people who do not have a stable job and their expectations of future employment and income, thus reducing their consumption of goods and services (Bande & Riveiro, 2013; Benito, 2006; Ravn & Sterk, 2017). Likewise, during a recession, unemployment tends to last longer and precarious employment is more frequent (Heidenreich, 2015; Kretsos & Livanos, 2016). It leads to a more significant decrease in the economic resources of individuals who face work problems and, consequently, to an increase in their levels of deprivation (Layte et al., 2001). Due to the labour impact of the Great Recession in countries such as Spain and Portugal, together with the subsequent weak recovery, it is expected that the incidence of unemployment and employment precariousness on material deprivation may have increased in the last decade. In addition to all this, job uncertainty due to the Covid-19 pandemic must also be considered.

### Data and Methods

In this paper, we used the microdata from European Union Statistics on Income and Living Conditions (EU-SILC). This database, harmonised at the European level, provides annual information on individuals’ and households' income, employment, and living conditions (Eurostat, 2021). We selected the waves of 2012 (a year of the Great Recession period), 2016 (a year of the recovery period) and 2020 (the last year for which the EU-SILC provides information). For each year, the EU-SILC offers data at the individual level and at the household level in various files. To carry out the analysis we have merged these files into one for Spain and Portugal separately. Our unit of analysis is the household and we restricted our sample to households with children under 18 years of age, with the final sample being 11,670 households in Spain and 7149 in Portugal.

In terms of child poverty, we focused our analyses on the measure of children's exposure to household material deprivation proposed by Ferrão et al. (2021). Although the EU-SILC provides information on specific child deprivation indicators, these indicators are only included in the waves of 2009 and 2014. In our dependent variable, we used the standard household material deprivation items that Ferrão et al. (2021) identify as those that could have a greater impact on the well-being of children, since the information on these items is provided annually by the EU-SILC. These items indicate whether (a) the dwelling is overcrowded; (b) the dwelling is dimly lit; (c) the home cannot be kept adequately warm; (d) there are arrears on mortgage, rent payments, utility bills, purchase instalments, or other loans^1^; (e) the household cannot afford a meal with meat, chicken, or fish every second day; (f) the household does not have a computer because it cannot afford it; (g) the household does not have a car because it cannot afford it; (h) there is noise from neighbours or the street; (i) there is pollution, grime, or other environmental problems in the area; and (j) there is crime, violence, or vandalism in the area. Specifically, the dependent variable is a dummy variable that takes the value 1 if the household experiences deprivation in at least 3 of the ten selected items and 0 otherwise. Setting the deprivation threshold at 3 or more items is a common practice at the European level (Bedük, 2018). In fact, this threshold is used in the official measures of household material deprivation and child deprivation of the European Union (Chzhen & Bradshaw, 2012; Guio, 2009; Guio et al., 2020) as well as in the studies by Bedük (2018) and Townsend (1979).

The key independent variable considered identifies households with employment insecurity, that is households where no one has a secure job. Similar to Bentley et al. (2019), this employment insecurity variable takes the value 1 if all the household members active in the labour market are unemployed, have a temporary job or are self-employed without employees. On the contrary, the variable takes the value 0 if at least one of the household members has a permanent job or is self-employed with employees. Eichhorst and Tobsch (2017) stated that these last two forms of employment are associated with a lower risk of precariousness and greater perceived job security.

The independent control variables used include parental and household characteristics. Specifically, we include dummy variables that indicate whether the parents of the household have a low educational level (1 = both parents have an educational level of ISCED 2 or lower; 0 = the father and/ or mother have an educational level of ISCED 3 or higher), whether the parents are from a foreign country (1 = both parents are from a foreign country; 0 = the father and/or mother are not from a foreign country), and whether the parents are young (1 = both parents are under 30 years of age; 0 = the father and/or mother are 30 years of age or older). We also consider the number of children (1 = three or more children in the household; 0 = one or two children in the household), the family structure (1 = only one parent lives in the household; 0 = both parents live in the household), the age of the youngest child in the household (1 = the youngest child is between 12 and 17 years of age; 0 = the youngest child is under 12 years of age), and the household equivalised disposable income (in thousands of euros). It should be noted that we control for the household income of 2011, 2015 and 2019 because the EU-SILC income data corresponds to the year before the survey. For this reason, we cannot take into account the effect of the pandemic crisis on household income, since the information for this variable in the 2020 data file is that of the income obtained by households in 2019.

The main descriptive statistics for all variables in each of the study years are reported in Tables 1 (Spain) and Table 2 (Portugal).

**Table 1:** Descriptive statistics by year, Spain

	2012				2016				2020			
	Mean	SD	Min	Max	Mean	SD	Min	Max	Mean	SD	Min	Max
Dependent variable												
Material deprivation	0.108	0.311	0	1	0.123	0.329	0	1	0.166	0.372	0	1
Independent variables												
Household employment insecurity	0.319	0.466	0	1	0.320	0.467	0	1	0.250	0.433	0	1
Low educated parents	0.292	0.455	0	1	0.257	0.437	0	1	0.210	0.407	0	1
Immigrant parents	0.142	0.349	0	1	0.095	0.293	0	1	0.120	0.324	0	1
Parents under 30 years	0.031	0.173	0	1	0.028	0.164	0	1	0.021	0.144	0	1
Three or more children in the household	0.074	0.261	0	1	0.077	0.266	0	1	0.084	0.278	0	1
Single-parent family	0.075	0.263	0	1	0.089	0.285	0	1	0.090	0.287	0	1
Youngest child 12 to 17 years of age	0.256	0.436	0	1	0.265	0.441	0	1	0.305	0.460	0	1
Household income	14.775	10.455	− 10.692	157.559	14.786	10.509	− 9.760	135.211	16.556	10.326	− 3.657	180.501

*Source* EU-SILC 2012, 2016 and 2020

Weighted data

**Table 2:** Descriptive statistics by year, Portugal

	2012				2016				2020			
	Mean	SD	Min	Max	Mean	SD	Min	Max	Mean	SD	Min	Max
Dependent variable												
Material deprivation	0.165	0.371	0	1	0.152	0.359	0	1	0.094	0.292	0	1
Independent variables												
Household employment insecurity	0.204	0.403	0	1	0.176	0.381	0	1	0.133	0.340	0	1
Low educated parents	0.534	0.499	0	1	0.398	0.490	0	1	0.261	0.439	0	1
Immigrant parents	0.032	0.177	0	1	0.018	0.133	0	1	0.021	0.145	0	1
Parents under 30 years	0.059	0.236	0	1	0.044	0.204	0	1	0.030	0.170	0	1
Three or more children in the household	0.045	0.207	0	1	0.048	0.215	0	1	0.054	0.226	0	1
Single-parent family	0.102	0.303	0	1	0.110	0.313	0	1	0.128	0.335	0	1
Youngest child 12 to 17 years of age	0.292	0.455	0	1	0.294	0.455	0	1	0.339	0.473	0	1
Household income	9.476	6.977	0.167	78.906	9.987	6.987	0.124	169.701	12.396	7.726	0.157	75.499

*Source* EU-SILC 2012, 2016 and 2020

Weighted data

Regarding the analytical plan, we start by showing the percentages of households with employment insecurity in each year of study in Spain and Portugal. In addition, we also show the percentages of fathers and mothers active in the labour market according to their employment status. Thus, we can see the employment situation of families with children in 2012, 2016 and 2020. Secondly, we estimate two logistic regression models for the samples of households with children from Spain and Portugal. In the first model, we analyse the relationship of the independent variable of employment insecurity with the variable of material deprivation. We also include the rest of the control explanatory variables and year dummies.^2^ The model is specified as follows:

$${\text{log }}\left[ {p_{i} / \, \left( {{1} - p_{i} } \right)} \right] \, = \beta_{0} + \beta_{{1}} E_{i} + \beta_{{2}} C_{i} + \beta_{{3}} D_{t}$$

where *p* is the probability that household *i* experiences deprivation; *E*_i_ represents the variable of employment insecurity; *C*_i_ is the set of control variables; and *D*_*t*_ represents the year dummies.

The second model includes interaction terms between the variable of employment insecurity and the year dummies (*E*_*i*_*D*_*t*_):

$${\text{log }}\left[ {p_{i} / \, \left( {{1} - p_{i} } \right)} \right] \, = \beta_{0} + \beta_{{1}} E_{i} + \beta_{{2}} C_{i} + \beta_{{3}} D_{t} + \beta_{{4}} E_{i} D_{t}$$

Whit this last model we can assess whether the association between household employment insecurity and the dependent variable has varied between 2012 and 2020.^3^

## Results

The descriptive findings in Table 3 show that the percentage of households with children where no one has secure employment was higher in Spain than in Portugal during the three years of the study.^4^ In the Spanish case, the percentage of households with employment insecurity in 2016 was very similar to that of 2012 (around 32% in the two years). However, it decreased to 25% in 2020. In Portugal, the data show a decrease in 2016 and, especially, in 2020.

**Table 3:** Percentage of households experiencing employment insecurity

	Spain	Portugal
2012	31.93	20.36
2016	32.01	17.64
2020	25.03	13.32

*Source* EU-SILC 2012, 2016 and 2020

The percentages are based on the total number of households with children between 0 and 17 years old

Tables 4 and 5 show the percentages of fathers and mothers active in the labour market according to employment status in Spain and Portugal, respectively. Focusing first on Spain, the data in Table 4 show that the percentages of fathers and mothers with a secure job (permanent employment or self-employed with employees) hardly changed between 2012 and 2016. In the same period, the percentages of unemployed fathers and mothers decreased. In contrast, the percentages of fathers and mothers with a temporary job or who are self-employed without employees increased. Data from 2020 indicate an improvement in the general employment situation of fathers and mothers compared to 2016.

**Table 4:** Paternal and maternal employment status, Spain

	Permanent job	Self-employed with employees	Temporary job	Self-employed without employees	Unemployed
% Fathers					
2012	53.53	5.70	11.01	9.37	20.39
2016	54.27	6.05	13.46	10.92	15.30
2020	61.12	5.83	12.35	9.76	10.94
% Mothers					
2012	47.76	2.76	13.18	4.81	31.50
2016	47.14	2.48	17.82	7.04	25.52
2020	56.13	2.12	13.71	6.54	21.49

*Source* EU-SILC 2012, 2016, and 2020

The percentages are based on the total number of fathers or mothers (as applicable) active in the labour market

**Table 5:** Paternal and maternal employment status, Portugal

	Permanent job	Self-employed with employees	Temporary job	Self-employed without employees	Unemployed
% Fathers					
2012	65.02	4.96	7.94	8.09	13.98
2016	67.82	4.44	10.90	7.09	9.77
2020	73.03	3.60	8.92	7.83	6.63
% Mothers					
2012	62.16	1.28	11.18	4.52	20.86
2016	61.81	2.33	12.99	6.44	16.43
2020	69.31	1.78	8.88	6.02	14.01

*Source:* EU-SILC 2012, 2016, and 2020

The percentages are based on the total number of fathers or mothers (as applicable) active in the labour market

From the information in Table 5, it stands out that between 2012 and 2016, the percentages of fathers with permanent or temporary employment rose in Portugal. In addition, the percentage of unemployed fathers decreased between these two years. Regarding mothers’ employment status, the main variation between 2012 and 2016 is reflected in the increase in temporary employment and self-employment without employees and the decrease in unemployment. As in Spain, the employment situation of both fathers and mothers in Portugal was better in 2020 than in 2016.

The results of the estimations of the logistic regression models are reported in Table 6. Focusing on model 1, we can observe that the coefficient of the household employment insecurity variable is positive and statistically significant in Spain and Portugal. This result shows that the likelihood of material deprivation in households with children is higher in households with employment insecurity than in those where at least one of the adult members has secure employment. Therefore, employment security seems to be a key factor in avoiding material deprivation in families with children. Most of the independent control variables also have a statistically significant association with the measure of material deprivation. In both countries, the risk of deprivation in households with children is higher if the parents have a low level of education, are immigrants or are under 30 years of age. In addition, in households with more than two children and those headed by single parents, the risk of deprivation is also greater. It can also be confirmed that the lower the household income, the greater the likelihood of material deprivation. The age of the youngest child is the only control variable that is not significantly associated with the dependent variable. Regarding the years dummies, the results differ between the two countries. In Spain, the risk of deprivation in households with children appears to be higher in 2016 and 2020 than in 2012. However, the coefficients of the year dummies are not statistically significant in the Portuguese case.

**Table 6:** Logistic regression of material deprivation in households with children

	Spain		Portugal	
	(1)	(2)	(1)	(2)
Household employment insecurity	0.535***	0.315**	0.523***	0.387**
Control variables				
Low educated parents	0.568***	0.568***	0.778***	0.778***
Immigrant parents	0.855***	0.857***	0.712***	0.713***
Parents under 30 years	0.457***	0.462***	0.792***	0.784***
Three or more children in the household	0.433***	0.430***	0.693***	0.699***
Single-parent family	0.348***	0.340***	0.188*	0.175
Youngest child 12 to 17 years of age	− 0.024	− 0.026	− 0.007	− 0.005
Household income	− 0.069***	− 0.069***	− 0.111***	− 0.111***
Years				
2016	0.304***	0.137	0.101	0.082
2020	0.790***	0.649***	0.006	− 0.114
Interactions				
Household employment insecurity*2016		0.319**		0.059
Household employment insecurity*2020		0.284*		0.393*
Constant	− 2.111***	− 1.995***	− 1.555***	− 1.511***
Observations	11,670	11,670	7149	7149
Pseudo R^2^	0.137	0.138	0.131	0.132

*Source* EU-SILC 2012, 2016 and 2020

***p < 0.01, **p < 0.05, *p < 0.10

**Fig. 3:**
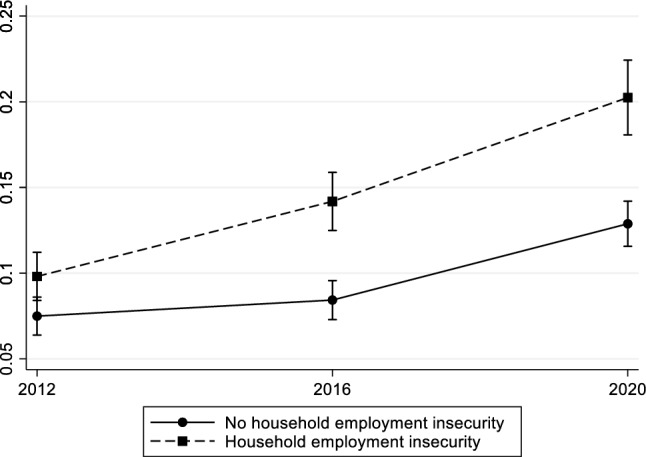
Predicted probabilities for the interaction between the year dummies and the household employment insecurity variable, Spain. *Source* Based on Table 6 (model 2, Spain)

The results of model 2 for Spain show that the coefficients of the interactions between the household employment insecurity variable and the years dummies are significant and with a positive sign. To interpret these interactions, we compute and plot predicted probabilities of material deprivation for households with and without employment insecurity in 2012, 2016, and 2020. Figure 3 shows that the predicted probabilities of deprivation increased in 2016 and specially in 2020. It should be noted that this increase has been greater in households with employment insecurity, so the differences with respect to households where at least one adult has secure employment seem to have increased.

**Fig. 4:**
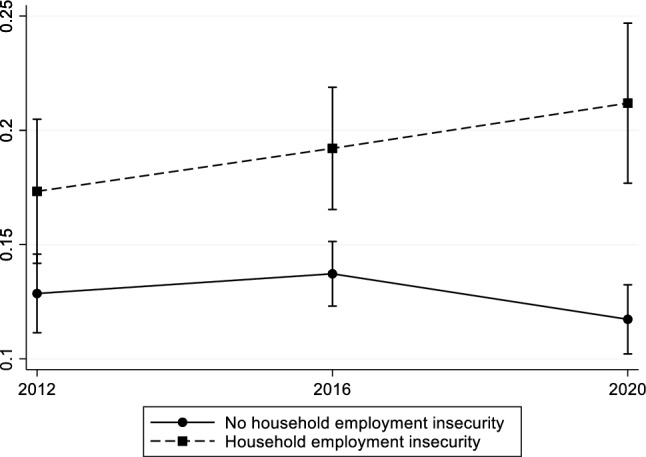
Predicted probabilities for the interaction between the year dummies and the household employment insecurity variable, Portugal. *Source* Based on Table 6 (model 2, Portugal)

Regarding the Portuguese case, the results of model 2 show that the coefficient of the interaction between the household employment insecurity variable and the 2016 dummy is not statistically significant, while the coefficient of the interaction with the 2020 dummy is significant and positive. As shown in Fig. 4, the differences in the predicted probabilities of material deprivation between the two types of households was roughly the same in 2012 and 2016. On the contrary, in 2020 the difference increased since the probability of deprivation in households with employment insecurity was higher than in 2016, while in households where at least one member had secure employment was lower.

## Discussion and Conclusion

In this article, we examine how the employment situation of families with children has evolved in the post-Great Recession period in Spain and Portugal and the relationship between household employment insecurity and children's risk of exposure to material deprivation. To do so, we use the EU-SILC data for the years 2012, 2016 and 2020.

Our descriptive analyses show that in the three years of study, employment insecurity in households with children has been lower in Portugal than in Spain. This is explained by the greater labour impact of the 2008 crisis in Spain, especially in terms of increased unemployment (Gutiérrez, 2014). It should also be taken into account that there is a greater integration of women in the labour market in Portugal, which makes households less vulnerable to unemployment (Rubery, 2013; Távora & Rodríguez-Modroño, 2018). The descriptive analyses also show that the recovery of employment security after the crisis was faster for Portuguese families. In 2016, the percentage of families with employment insecurity in Portugal was lower than in 2012, while this decrease was not observed in Spain. The results of the employment situation of parents at the individual level indicate that, although in Spain the percentage of unemployed fathers and mothers in 2016 was lower than in 2012, this decrease in unemployment was principally due to the increase in temporary employment and self-employed without employees. In Portugal, although an increase in job precariousness also accompanied the decrease in unemployment, the percentage of fathers active in the labour market with permanent employment increased slightly between 2012 and 2016, which may explain the reduction in the percentage of households with employment insecurity. Perhaps an issue to be studied in the future is whether the measures to achieve greater flexibility in employment after the 2008 crisis not only resulted in an increase in job precariousness but also facilitated the entry into the labour market of many women such as those with children. Finally, Spanish and Portuguese families had a better employment situation in 2020, both at the household level and at the individual level, compared to 2016. This result may seem contradictory given the negative consequences of the Covid-19 pandemic on the labour market. It is necessary to consider the great impact of the 2008 crisis in the countries of southern Europe and the slow recovery that followed (OECD, 2018). Likewise, the measures adopted to retain employment during the pandemic caused the increase in unemployment to be much less than that of the Great Recession (ILO, 2020).

Secondly, the findings of this research indicate that employment insecurity is associated with a greater likelihood of material deprivation in households with children. These results are similar to those of previous studies showing that unemployment and job precariousness increase the risk of material deprivation (Álvares & Amaral, 2014; Cheung et al., 2019; de Graaf-Zijl & Nolan, 2011; Eamon & Wu, 2011). On the other hand, one of the main findings of our analyses is that the differences in the risk of material deprivation between households with and without employment insecurity have increased in recent years. Specifically, we find that in Spain, these differences were greater in 2016 and 2020 than in 2012. Focusing on the increase in 2016, the main reasons may be the economic deterioration suffered by families in the crisis period and the subsequent slow recovery of the labour market. All this could reduce the accumulated resources of many families that faced employment problems, thus increasing the risk of deprivation (Layte et al., 2001). Regarding the increase in 2020, this may be due to the combination of the slow recovery after the Great Recession and job uncertainty due to the Covid-19 pandemic. The uncertainty could especially affect people who did not have a stable job, which reduced their consumption of goods necessary for families’ well-being (Bande & Riveiro, 2013; Benito, 2006; Ravn & Sterk, 2017). In Portugal, there was no significant change in the relationship between household employment insecurity and material deprivation between 2012 and 2016. The lower impact of the crisis in terms of unemployment and the better integration of mothers in the labour market could have caused the accumulated resources of families to decrease less than in Spain. However, despite the effect of the pandemic on the labour market was not as great as that of the Great Recession, the job uncertainty in 2020 does seem to increase the risk of deprivation in households where none of the members has a steady job. Therefore, we can conclude that the employment context of a country is essential in the relationship between employment status and the risk of material deprivation in families with children.

In general, the results of this research show that after the Great Recession, the employment situation of Spanish and Portuguese families improved. Despite this, the economic deterioration suffered by many families in the crisis period, the subsequent slow recovery of the labour market and, lastly, the job uncertainty due to the Covid-19 pandemic have increased the risk of child exposure to household material deprivation in those households where there is no employment security. In other words, the risk of deprivation associated with employment insecurity has increased over the last ten years. These circumstances can negatively affect the well-being of the children who live in these households and, therefore, their future development (Duncan et al., 2012; Lacour & Tissington, 2011; Oshio et al., 2010). Several studies point to the detrimental effect of economic and material hardships on family interactions and functioning (Bao & Greder, 2022; Conger et al., 1994; Liu et al., 2022). In this sense, our findings contribute to the identification of Spanish and Portuguese households where these problems have been amplified in recent years. Consequently, social policy intervention would be very timely to minimize the harmful effects of job loss and job precariousness on families with children.

One of the conclusions obtained from the comparative analysis of Spain and Portugal is the relevance of women's participation in the labour market since this can reduce employment insecurity in families with children and offer greater protection in contexts of economic and labour difficulties. Although there have been greater advances in family policies in Spain, compared to countries such as Italy or Greece, and the integration of women in the labour market is increasing, improvements are still needed in policies for reconciling employment and family life (Escobedo & Wall, 2015).

Future research should analyse in more detail the labour and economic consequences that the Covid-19 pandemic may have had on families. Our results show a large initial impact on material deprivation in those households with children in which none of the adult members has secure employment. Studies need to take into account the longer-term effect of the pandemic and subsequent recovery.

## Data Availability

The data that support the findings of this study have to be provided by Eurostat.

## Appendix

See Appendix Tables

**Table 7:** Logistic regression of material deprivation in households with children by year

	Spain			Portugal		
	2012	2016	2020	2012	2016	2020
Household employment insecurity	0.294**	0.544***	0.665***	0.558***	0.323**	0.808***
Control variables						
Low educated parents	0.616***	0.630***	0.456***	0.651***	0.740***	0.948***
Immigrant parents	0.533***	0.934***	0.997***	1.091***	0.697**	0.019
Parents under 30 years	0.633**	0.079	0.730***	0.926***	0.417*	1.186***
Three or more children in the household	0.510***	0.495***	0.329**	0.962***	0.611***	0.460*
Single-parent family	0.226	0.336**	0.389***	0.178	0.198	0.146
Youngest child 12 to 17 years of age	− 0.049	− 0.028	− 0.011	0.046	− 0.079	0.056
Household income	− 0.074***	− 0.082***	− 0.059***	− 0.053***	− 0.157***	− 0.100***
Constant	− 1.913***	− 1.707***	− 1.491***	− 1.946***	− 1.003***	− 1.827***
Observations	3638	3881	4151	1630	2871	2648
Pseudo R^2^	0.106	0.155	0.137	0.093	0.145	0.145

*Source* EU-SILC 2012, 2016 and 2020

***p < 0.01, **p < 0.05, *p < 0.10

^7^ and

**Table 8:** AROPE components for the subsample of study households

	Spain			Portugal		
	2012	2016	2020	2012	2016	2020
Severe material deprivation	7.23	6.80	8.42	9.49	9.01	3.13
Risk of poverty	25.99	27.41	25.15	19.66	20.79	16.68
Low work intensity	10.51	10.11	5.07	5.80	4.98	2.67

*Source* EU-SILC 2012, 2016 and 2020

^8^.

## Footnotes

1 As in the official material deprivation measure for the European Union, we group the items of arrears on mortgage or rental payments (hs011), arrears on utility bills (hs021), and arrears on hire purchase instalments or other loan payments (hs031).

2 Additionally, Table 7 in the appendix shows the results of this model estimated for each year separately.

3 To identify the existence of multicollinearity issues between the different independent variables included in the models, we used the variance inflation factor (VIF). Since no variable has a VIF value greater than 10, multicollinearity does not seem to be a problem. We also estimated alternative models, available on request, in which we remove the control variables (one in each model). The main results remain unchanged.

4 Apart from these descriptive analyses, we present the AROPE indicators for our study household samples in Table 8 of the appendix.
